# Supplementary material for: Many-molecule encapsulation by an icosahedral shell
Source: eLife. 2016 May 11;5:e14078. doi: 10.7554/eLife.14078 (PMC4947392; doi:10.7554/eLife.14078)
Supplement: Figure 3—source data 2. — The sizes of each cargo globule and shell assemblage, and associations between shell assemblages and cargo globules, were determined by clustering. The outcome was then categorized according to the criteria listed in this table. DOI: http://dx.doi.org/10.7554/eLife.14078.011 [file elife-14078-fig3-data2.zip › Figure 3—source data 2.pdf]

Symbol	Name	Description
■	Complete capsid (full)	Shell comprises 12 pentamers and 20 hexamers, with more than 50 cargo molecules inside
◆	Complete capsid (empty)	Shell comprises 12 pentamers and 20 hexamers, with fewer than 50 cargo molecules inside
●	Attached	Number of cargo molecules in the globule>150, 4 or fewer shells attached to the globule, each with 10 to 31 subunits
✳	Over-nucleated/Malformed	Number of cargo molecules in largest globule<150, number of subunits in one or more shells >32 or more than 12 pentamers in a shell, or more than 20 hexamers in a shell, or more than 4 shells with fewer than 10 subunits attached to the globule
×	Stalled	Same as Over-nucleated, except with more than 150 cargo molecules in the globule
□	Globule	Number of cargo molecules in the globule <50, number of subunits in the largest shell<10
⊙	Unnucleated	Number of cargo molecules in the globule <50, number of subunits in the largest shell<10
